# *SDHA*-related phaeochromocytoma and paraganglioma: review and clinical management

**DOI:** 10.1530/ERC-24-0111

**Published:** 2024-09-21

**Authors:** Adam I Kaplan, Trisha Dwight, Catherine Luxford, Diana E Benn, Roderick J Clifton-Bligh

**Affiliations:** 1 Kolling Institute of Medical Research, The University of Sydney, Sydney, Australia; 2 Department of Endocrinology, Royal North Shore Hospital, Sydney, Australia; 3 Cancer Genetics Laboratory, Kolling Institute, Royal North Shore Hospital, Sydney, Australia; 4 Faculty of Medicine and Health, The University of Sydney, Sydney, Australia

**Keywords:** hereditary, neoplastic syndromes, paraganglioma, pathogenic variant, phaeochromocytoma, pseudohypoxia, SDHA, succinate dehydrogenase subunit-A

## Abstract

Phaeochromocytomas and paragangliomas (collectively termed PPGL) are rare yet highly heritable neuroendocrine tumours, with over one-third of cases associated with germline pathogenic variants (PVs) in numerous genes. PVs in the succinate dehydrogenase subunit-A gene (*SDHA*) were initially implicated in hereditary PPGL in 2010, and *SDHA* has since become an important susceptibility gene accounting for up to 2.8% of cases. However, it remains poorly understood, particularly regarding the clinical nature of *SDHA* PPGL, rates of recurrence and metastasis, and the nature of metastatic disease. We present a narrative review of *SDHA-*related PPGL, covering pathophysiology, relevance to current clinical practice, and considerations for clinical genetics. We analyse a pool of 107 previously reported cases of *SDHA-*associated PPGL to highlight the spectrum of *SDHA*-related PPGL. Our analysis demonstrates that *SDHA* PPGL occurs across a wide age range (11–81 years) and affects men and women equally. *SDHA* PPGL typically presents as single tumours (91%), usually occurring in the head and neck (46%) or abdomen (43%, including 15% with phaeochromocytomas). Metastatic disease was reported in 25.5% of cases, with bone (82%) and lymph nodes (71%) being the most common sites of metastasis, often identified many years after the initial diagnosis. A family history of *SDHA-*related neoplasia was rare, reported in only 4% of cases. Understanding the clinical nature and risks associated with *SDHA* PVs is essential for facilitating the optimal management of patients and their families.

## Introduction

Phaeochromocytomas and paragangliomas (collectively termed PPGL) are rare neuroendocrine tumours originating from chromaffin cells of the adrenal medulla, and neuroendocrine paraganglia, respectively. Paragangliomas (PGLs) are subdivided into sympathetic and parasympathetic (Benn *et al.* 2015): sympathetic PGLs arise from extra-adrenal chromaffin cells in the abdomen, pelvis, or posterior mediastinum and typically secrete catecholamines, whereas parasympathetic PGLs arise from parasympathetic ganglia in the head and neck or the anterior and middle mediastinum and are often biochemically silent and indolent (Timmers *et al.* 2024). Phaeochromocytomas are catecholamine-secreting tumours confined to the adrenal medulla (Lenders *et al.* 2014).

PPGL are highly heritable, with up to 40% of cases associated with germline pathogenic variants (PVs) in one of over 20 genes (*EGLN1, FH, KIF1B, KMT2D, MAX, MDH2, MERTK, MET, NF1, RET, SDHA, SDHAF2, SDHB, SDHC, SDHD, TMEM127, VHL, SLC25A11, GOT2, IHD3B, DNMT3A, DLST,SUCLG2*), and genetic testing is recommended for all patients with PPGL (Lenders *et al.* 2014, Plouin *et al.* 2016). PVs associated with PPGL occur most commonly in the succinate dehydrogenase (SDH) subunit genes: *SDHA, SDHB, SDHC, and SDHD*.

Heterozygous germline *SDHA* PVs are associated with an increased risk of neoplasms, with *SDHA-*related PPGL being the focus of this review. Germline *SDHA* PVs with tumour loss of SDHA expression on immunohistochemistry have additionally been identified in pituitary neuroendocrine tumours/adenomas (Dwight *et al.* 2013*a*), neuroblastoma (Dubard Gault *et al.* 2018), and gastrointestinal stromal tumours (GISTs) (Dwight *et al.* 2013*b*); *SDHA* PVs account for approximately half of all SDH-deficient GISTs (Pantaleo *et al.* 2022). Somatic *SDHA* PVs with the associated loss of SDHA immunohistochemistry staining have also been demonstrated with renal cell carcinoma (Yakirevich *et al.* 2015).

The causal link between germline PVs in *SDHB, SDHC,* and* SDHD* and development of PPGL is well established (Baysal *et al.* 2000, Niemann & Muller 2000), with *SDHB* PVs associated with a higher risk of metastasis (Astuti *et al.* 2001). *SDHA* germline PVs were more recently associated with PPGL, with the first case identified in 2010 (Burnichon *et al.* 2010). Since then, an increasing number of studies have reported *SDHA* PVs in PPGL. However, understanding the clinical nature of *SDHA* disease, rates of penetrance and malignancy, and the pathogenicity of certain variants remains uncertain*.* Hence, consensus management of patients with *SDHA* PVs has been less defined than other *SDH* genes.

This narrative review summarises the spectrum of *SDHA-*related PPGL disease, with a focus on the translational relevance of *SDHA* research for the contemporary clinical management of patients and their families with *SDHA* PVs. We have collated 107 patients with *SDHA*-related PPGL previously reported in the literature and summarise the current knowledge regarding *SDHA* PPGL disease.

## Pathophysiology

### Succinate dehydrogenase (SDH)

Succinate dehydrogenase (SDH) is a key enzyme in the process of oxidative phosphorylation in eukaryotes. It has a unique dual role in both the Krebs cycle, where it catalyses the conversion of succinate to fumarate, and in the Electron Transport Chain (ETC), where it constitutes complex II, which transfers electrons to ubiquinone. SDH is a heterotetrameric protein, with all four subunits encoded by nuclear genes. The catalytic site is composed of the *SDHA* and *SDHB* subunits, which are hydrophilic and project into the mitochondrial matrix. The other two subunits, *SDHC* and *SDHD*, are hydrophobic and anchor the complex to the inner mitochondrial membrane, as well as provide the site for ubiquinone binding. *SDHA*, the focus of this review, is the major catalytic subunit of SDH. It contains a covalently attached flavin adenine dinucleotide (FAD) prosthetic group, which is reduced by the reaction of succinate to fumarate, becoming FADH_2_. Electrons generated by this enzymatic reaction are transferred from FADH_2_ to Fe-S moieties in *SDHB*, and eventually to ubiquinone (Q) by *SDHC* and *SDHD* as part of the ETC (Rutter *et al.* 2010).

### *SDHA* and tumorigenesis

*SDHA*, like the other *SDHx* genes, is a classical tumour suppressor gene, wherein biallelic *SDHA* inactivation is associated with oncogenesis (Burnichon *et al.* 2010). SDH deficiency arising from the loss of any SDH subunit leads to decreased SDH activity, resulting in a common biochemical mechanism of tumorigenesis for *SDHx* variants. SDH deficiency leads to at least two specific downstream consequences. Firstly, decreased electron flow through the mitochondrial respiratory chain leads to increased reactive oxygen species (ROS) such as superoxides, which are known drivers of proliferation in various forms of cancer (Bénit *et al.* 2022). Secondly, succinate accumulation inhibits several α-ketoglutarate-dependent dioxygenases, which leads to hypoxia-inducible factor alpha (HIF-α) stabilisation and DNA hypermethylation (Moog *et al.* 2020).

Succinate inhibition of prolyl hydroxylase (PHD, also known as EGLN) prevents physiological degradation of HIF-α via von Hippel-Lindau-mediated polyubiquitination; HIF-α then regulates specific target genes involved in adaptation and proliferation under hypoxic conditions, including vascular endothelial growth factor, VEGF (Bao *et al.* 2021). This activation of the hypoxic response in the absence of hypoxia is termed ‘pseudo-hypoxia’. Hence, *SDHx*-associated PPGLs are defined within the pseudohypoxia group (also referred to as cluster 1) of PPGL.

Succinate inhibition of ten-eleven translocation (TET) hydroxylases leads to DNA demethylation, with their inhibition resulting in global hypermethylation in SDH-deficient cells (Xiao *et al.* 2012). As methylation of histone and DNA are key factors regulating gene expression, they may contribute to tumorigenesis.

In summary, SDHA deficiency caused by *SDHA* PVs results in succinate accumulation, which inhibits numerous α-ketoglutarate-dependent dioxygenases, leading to HIF activation and hypermethylation of target genes, promoting angiogenesis and pro-oncogenic pathways.

### Identification of *SDHA* as a PPGL gene

*SDHA* was first linked with hereditary paraganglioma/phaeochromocytoma in 2010 when Burnichon *et al.* (2010) identified a patient with abdominal PGL who had a heterozygous germline missense variant in *SDHA*, c.1765C>T p.(Arg589Trp). The following year, the same research group published results in which they identified germline *SDHA* variants in 3% of apparently sporadic PPGL (6 out of 198 cases), with all cases involving loss of heterozygosity (LOH) and negative SDHA immunohistochemistry (Korpershoek *et al.* 2011). Since then, many more cases of *SDHA* PVs in PPGL have been reported. In two of the largest studies to date, 3–7.6% of genetically unexplained PPGL cases were found to carry germline *SDHA* variants, with 44–70% of the *SDHA* cases presenting with head and neck PGL (HNPGL) (Bausch *et al.* 2017, van der Tuin *et al.* 2018). A recent study found *SDHA* PVs in 2.8% of 1727 patients who underwent multigene panel testing due to suspicion of hereditary PPGL (Horton *et al.* 2022).

## Clinical spectrum of *SDHA-*related PPGLs – analysis of 107 cases previously reported in the literature

As a comparatively novel PPGL-associated gene with low disease penetrance, reports of *SDHA-*associated PPGL have thus far been limited to relatively small, isolated case series, which has constrained our understanding of the clinical spectrum of *SDHA* PPGL disease.

We collated 107 reported cases of *SDHA-*related PPGL (for methods, please refer to the Supplementary Material; see the section on supplementary materials given at the end of this article; Welander *et al*. 2013, Currás-Freixes, *et al*. 2015, von Dobschuetz *et al*. 2015, Casey *et al*. 2017*a*, *b*). There were 38 different germline variants in our case series, the most common of which was c.91C>T p.(Arg31*), which accounted for 48/98 (49%) of PPGL cases. The pathogenic status of the *SDHA* variants is outlined in Table 1. Ninety-eight cases involved pathogenic or likely pathogenic *SDHA* variants (primary analysis), and 9 cases involved *SDHA* variants of uncertain significance (VUS), which were analysed separately (information on individual PPGL cases can be found in Supplementary Table 1).

**Table 1:** *SDHA* variants from 107 *SDHA-*related PPGL cases collated from the literature.

*SDHA* variant	Varsome analysis^a^	ClinVar status
c.1A>C	P (17P-0B)(PVS1, PP5, PM2)	Pathogenic
c.1A>T	P (17P-0B)(PVS1vstrong, PP5vstrong, PM2sup)	Pathogenic/likely pathogenic
c.2T>G	P (17P-0B)(PVS1vstrong, PP5vstrong, PM2sup)	Pathogenic/likely pathogenic
c.3G>C	P (11P-0B)(PVS1, PP5, PM2)	Pathogenic
c.5’UTR_3’UTRdel	Not reported	Not reported in ClinVar
c.91C>T	P (17P-0B)(PVS1vstrong, PP5vstrong, PM2sup)	Pathogenic/likely pathogenic
c.223C>T	P (17P-0B)(PVS1vstrong, PP5vstrong, PM2sup)	Pathogenic/likely pathogenic
c.296A>G	LP (8P-0B)(PP3strong, PM5mod, PM2sup, PP2sup)	Uncertain significance
c.394T>C	LP (6P-0B)(PP3, PM2, PP5)	Not reported in ClinVar
c.457-1G>A	P (13P-0B)(PVS1vstrong, PP5strong, PM2sup)	Pathogenic/likely pathogenic
c.563G>A	LP (7P-0B)(PM5mod, PP3mod, PM2sup, PP2sup, PP5sup)	Pathogenic (1); Likely pathogenic (2); Uncertain significance (3)^b^
c.566G>A	LP (6P-0B)(PM5, PP3, PM2, PP2)	Not reported in ClinVar^c^
c.667delG	P (17P-0B)(PVS1vstrong, PP5vstrong, PM2sup)	Pathogenic/likely pathogenic
c.778G>A	P (18P-0B)(PP5vstrong, PS1strong, PP3strong, PM2sup, PP2sup)	Pathogenic/likely pathogenic
c.820G>A	LP (6P-0B)(PP3, PM2, PP2)	Not reported in ClinVar^c^
c.923C>T	LP (7P-0B)(PP3, PM2, PP2, PP5)	Likely pathogenic (3); Uncertain significance (2)
c.940G>A	LP (9P-0B)(PP3, PM2, PP2, PP5)	Likely pathogenic (1); Uncertain significance (1)
c.985C>T	P (13P-0B)(PVS1, PP5, PM2)	Pathogenic
c.1177G>A	LP (6P-0B)(PP3strong, PM2sup, PP2sup)	Uncertain significance (6); likely benign (1)
c.1283>1298del	Not reported	Not reported in ClinVar^c^
c.1316G>A	LP (6P-0B)(PP3strong, PM2mod, PP2sup)	Uncertain significance
c.1334C>T	LP (7P-0B)(PP3, PM2, PP2, PP5)	Likely pathogenic (2); uncertain significance (2)
c.1338delA	P (17P-0B)(PVS1, PP5, PM2)	Pathogenic
c.1340A>G	LP (8P-0B)(PP3strong, PS3sup, PM2sup, PP2sup, PP5sup)	Pathogenic (1); uncertain significance (3)
c.1361C>A	LP (6P-0B)(PP3strong, PM2sup, PP2sup)	Uncertain significance
c.1432_1432+1del	P (17P-0B)(PVS1vstrong, PP5vstrong, PM2sup)	Pathogenic/likely pathogenic
c.1534C>T	P (17P-0B)(PVS1vstrong, PP5vstrong, PM2sup)	PathogeniclLikely pathogenic
c.1753C>T	P (10P-0B)(PP3strong, PM1sup, PM5sup, PM2sup, PP5sup)	Pathogenic (2); Likely pathogenic (5); Uncertain significance (2)
c.1754G>A	P (10P-0B)(PP3strong, PM1, PM5, PM2, PP5)	Pathogenic (1); likely pathogenic (2); uncertain significance (2)
c.1765C>T	P (19P-0B)(PS3vstrong, PM5strong, PP3strong, PM1mod,PM2sup)	Pathogenic/likely pathogenic
c.1766G>A	P (19P-0B)(PP5, PM5, PP3, PM1, PM2)	Likely pathogenic
c.1795-3C>G	LP (6P-0B)(PP3, PP2, PP5)	Likely pathogenic (1); uncertain significance (3)
c.622T>C^d^	VUS (4P-0B)(PP3mod, PM2sup, PP2sup)	Uncertain significance
c.629G>A^d^	VUS (2P-0B)(PM2sup, PP2sup)	Uncertain significance
c.1115C>G^d^	VUS (4P-0B)(PP3mod, PM2sup, PP2sup)	Uncertain significance
c.1273G>A^d^	VUS (2P-2B)(PM2sup, PP2sup, BP4mod)	Uncertain significance
c.1799G>A^d^	VUS (1P-0B)(PP2sup)	Uncertain significance (7); likely benign (1)
c.1865G>A^d^	VUS (5P-0B)(PVS1, PM2)	Not reported in ClinVar

^a^Varsome classifications;^ b^Where ClinVar interpretation was conflicting, the various predicted effects with corresponding number of reports (X) have been shown; ^c^Not reported in ClinVar, however, the variants were reported as ACMG class 4 (likely pathogenic) or class 5 (pathogenic) in literature analysis (Bausch *et al.* 2017); ^d^The final six *SDHA* variants were identified as variants of uncertain significance (VUS). The 9 patients with these variants were analysed separately. P, pathogenic; LP, likely pathogenic; VUS, variant of uncertain significance.

### Primary analysis (*n* = 98)

**Figure 1:**
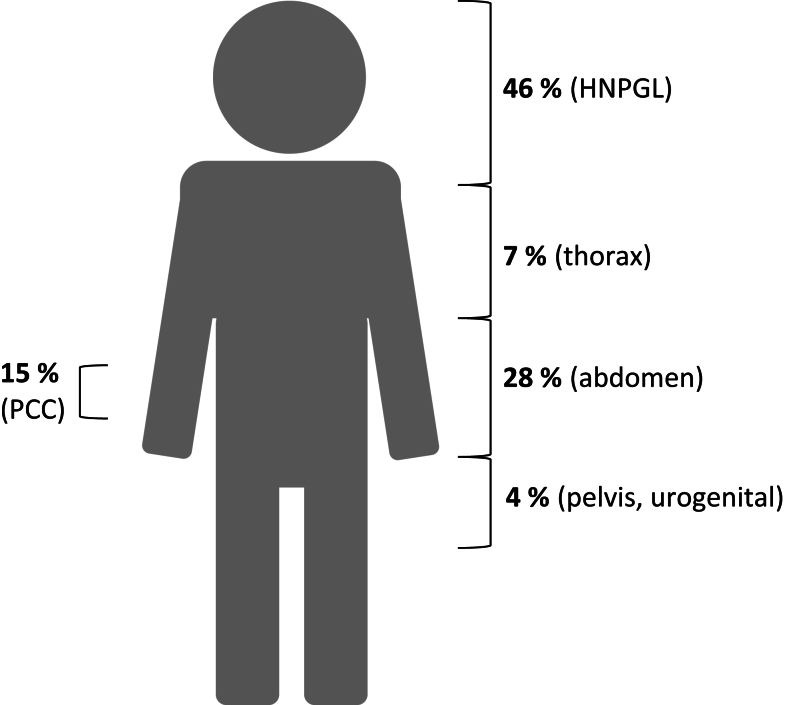
Distribution of PPGL disease in the pooled patient cohort.

Pooled data for the primary analysis are summarised in Table 2. Patients had a mean age of 40.0 years (y) (SD: 15.5) with a nearly equal sex distribution. The most common forms of PPGL disease were HNPGL (of which carotid body tumours were the most prevalent), abdominal PGL, and pheochromocytomas (Fig. 1). Most cases presented as single PPGL at diagnosis, although 9.2% presented with multiple primary lesions. Local recurrence was reported in some cases, with a median time to recurrence of 1.5 years (range: 2 months to 36 years). Metastases were identified in 25/98 cases (25.5%).

**Table 2:** Clinical features of 98 patients previously reported in the literature with *SDHA* PV-related pheochromocytoma (PCC) and paraganglioma (PGL).

Feature		*SDHA-*related PPGL
**Mean age at diagnosis,** y (SD)^a^		40.0 (15.5)
**Gender,**n/N (%)^b^	Male	50/98 (51%)
	Female	48/98 (49%)
**Single PGL at presentation,**(%)^b^		90.8
**Location,***n* (% of 103 PPGL^c^)	PCC	15 (15%)
	HNPGL - carotid	20 (19%)
	HNPGL - jugular	10 (10%)
	HNPGL - jugular tympanic	2 (1.9%)
	HNPGL - vagal	9 (8.7%)
	HNPGL - thyroid	2 (1.9%)
	HNPGL - other	4 (3.9%)
	TAPGL - thorax	7 (6.8%)
	TAPGL - abdomen	29 (28%)
	TAPGL - pelvis	2 (1.9%)
	TAPGL - other	2 (1.9%)
	PGL - unspecified	1 (0.97%)
**Immunohistochemistry,**n/N (%)^b^	SDHA negative	16/18 (89%)
	SDHB negative	16/16 (100%)
**Recurrent disease**	Local recurrence, *n*	13
	Median time to recurrence, years	1.5
**Metastatic disease,**n/N (%)^b^	Metastatic disease (overall)	25
	Synchronous presentation	7/25 (28%)
	Metachronous presentation	12/25 (48%)
	Median time to metachronous presentation, years	5.5
	Undefined presentation	6/25 (24%)
	Lymph node involvement	12/17 (71%)
	Bone involvement	14/17 (82%)
	Lung involvement	6/17 (35%)
	Liver involvement	2/17 (12%)
	Adrenal involvement	1/17 (6%)
**Biochemical status,** *n*	Biochemically active	35
	Biochemically silent	27
	Unknown profile	36
**Plasma catecholamine**^d^**,**n/N (%)^b^	Noradrenaline (NA)	28/35 (80%)
	Adrenaline (A)	4/35 (11%)
	Dopamine (DOPA) Unknown	10/35 (29%) 4/35 (11%)
**Family history of PGL/PCC or*****SDHA-*****related tumours**, n/N (%)^b^		4/98 (4%)

^a^ Age was known for 97 of the 98 patients analysed; ^b^ n/N (%): n represents the number of patients with the characteristic, while N represents the number of patients for whom the information was available. ^c^ Percentage of the 103 PPGL diagnosed at the various locations. The database included 98 patients; however, there were 103 PPGLs when adjusted for patients with multiple tumour types. ^d^ Ten cases of PPGL demonstrated elevations in multiple catecholamines. PCC, phaeochromocytoma; HNPGL, head and neck paraganglioma; TAPGL, thoracoabdominal paraganglioma; PGL, paraganglioma; SD, standard deviation.

Biochemical status was reported for 62 (63%) patients. Of these, 35 patients had biochemically active PPGL (56%), with noradrenaline (NA) secretion being most common, followed by dopamine, and then adrenaline. Ten patients demonstrated elevations in multiple catecholamines, and all four cases identified with elevated adrenaline concentrations also demonstrated elevation in noradrenaline, dopamine, or both. Only four patients had a known family history of PPGL or *SDHA-*associated neoplasms.

### Metastatic disease

In our analysis, metastatic disease was reported in 25/98 patients (25.5%). When case studies and case series solely reporting metastatic PPGL were excluded from analysis, the prevalence was 15% (13 of 86 cases).

Clinical details were available for 22 of the 25 metastatic cases. The mean age at primary diagnosis was 39.5 years (SD:16) for metastatic cases, with 15 of the 22 cases occurring in males (68%). Metastases occurred most commonly from thoracoabdominal PGLs (17 of 22 cases, 77%), followed by phaeochromocytoma (4 of 22 cases, 18%), and a single case occurred from a non-secreting vagal HNPGL. Details regarding biochemical activity were available for 19/25 metastatic cases: plasma or urinary norepinephrine/normetanephrine was increased in 17 of 19 cases (89%), either alone (*n* = 10), or in combination with elevated dopamine/methoxytyramine (*n* = 4) or epinephrine/metanephrine (*n* = 2), or both (*n* = 1). Plasma or urine dopamine or methoxytyramine was increased in 6 of 19 cases (32%).

Time course was reported in 19/25 metastatic cases: 12 (63%) presented with metastases at least 6 months after the initial diagnosis (metachronous), with a mean time to metastasis of 10 years following primary PPGL diagnosis. The most common sites of metastasis were bone, followed by lymph nodes, lung, liver, and adrenals (Table 2). Importantly, 11 of 13 (85%) patients with local recurrence developed metastases.

### Variants of uncertain significance (*n* = 9)

There were nine PPGL cases associated with *SDHA* VUS, which were analysed separately from the primary analysis. The clinical data for these cases are summarised in Supplementary Table 2. Similarly to the primary analysis, there was a near-equal gender split in patients; most patients presented with single PPGL, and the distribution of PPGL was similar, with HNPGL the most common, followed by PCC and abdominal PGL. There was one report of disease recurrence (12 years following the initial disease). Notably, there were no reports of metastasis in these patients.

## *SDHA*-related PPGL in clinical medicine

### Metastatic potential

Previous estimates for the prevalence of metastatic disease in *SDHA* PPGL vary considerably. Two recent reviews estimated prevalence at 20.56% and 16% (95% CI 4–51%) respectively (Jha *et al.* 2019, Lee *et al.* 2020). However, a 2019 review estimated metastatic risk with *SDHA* PPGL as high as 30–66%, second only to *SDHB* in terms of metastatic risk (Guha *et al.* 2019).

While our analysis suggests a more conservative estimate for metastatic risk, there are several limitations. Firstly, our analysis was limited to cases reported in the literature and, therefore, susceptible to positive publication bias. Secondly, there is possible selection bias, with 12 cases of metastasis presented in case reports or case series that focused on metastatic *SDHA* disease. While the largest of these studies (which accounted for 10 of these 12 cases) noted that their tertiary centre also cared for five cases of non-metastatic disease in the same period (resulting in a high overall metastatic prevalence of 66% in their single centre), it is possible that the inclusion of these studies inflates the estimated prevalence of metastatic disease. Conversely, there appears to be a significant delay to metastatic disease in many *SDHA* cases, which may, therefore, lead to an underestimate of the true rate of metastatic disease. Although larger studies are needed, *SDHA* PPGL should be regarded as having high metastatic potential, with metastasis often presenting years after the primary diagnosis, and often associated with recurrence. Current recommendations for follow-up of patients with germline *SDHA* PVs and a history of surgically resected PPGL are 6–12 monthly biochemical measures and 12–24 monthly imaging consisting of MRI and/or low-dose chest CT (Nölting *et al.* 2022).

### Genetic testing in current clinical practice

Genetic testing is recommended for all patients presenting with PPGL (Plouin *et al.* 2016). Genetic heterogeneity led previously to algorithms or protocols to prioritise genetic testing based on several factors, including biochemical signature, age at presentation, tumour location, presence of metastases, and family history (Lefebvre and Foulkes 2014). More recently, multigene panel, exome, or genome testing has become standard practice (Toledo *et al.* 2017). Nevertheless, technical challenges exist for testing *SDHA*, including the presence of three highly homologous *SDHA* pseudogenes (Benn *et al.* 2015).

Tumour SDHB immunohistochemistry (IHC) is a widely used technique that helps triage *SDHx* genetic testing (Gill *et al.* 2010, Castelblanco *et al.* 2013, Papathomas *et al.* 2015); as noted above, loss of SDHB staining occurs from genetic disruption of any of the *SDHx* genes. *SDHA* IHC is also useful when SDHB staining is negative since the loss of SDHA staining is usually associated with the discovery of an *SDHA* PV in either germline or tumour (Korpershoek *et al.* 2011, Benn *et al.* 2015).

### Identification of a germline *SDHA* variant in PPGL disease – implications for clinical management

The classification of a PPGL as *SDHA*-related has various implications for clinical management.

Preoperative identification of a germline PV allows preoperative risk analysis for metastatic potential and risk of recurrence and may influence the surgical approach in PPGL patients (Nockel *et al.* 2018). Specifically, patients with *SDHB* or *SDHA* variants were more likely to have received an open surgical approach compared to a minimally invasive approach and were more likely to have received a total rather than partial adrenalectomy (Nockel *et al.* 2018).

Early genetic testing in patients diagnosed with PPGL may result in improvements in patient management more generally. A recent multicentre retrospective study of 221 PPGL patients with *SDHB, SDHC, SDHD,* or *VHL* germline PVs demonstrated that patients who learnt of their genetic status within 12 months of their PPGL diagnosis received more thorough follow-up, were significantly more likely to remain in follow-up, any identified PPGL were smaller, and metastases less extensive, compared to patients who received genetic testing at least 7 years following the initial PPGL diagnosis (Buffet *et al.* 2019). While *SDHA* was not included in the study, it indicates potential benefits to patient management and outcomes with genetic testing.

In the future, an improved understanding of the risk of metastatic spread and tumour recurrence with *SDHA* variants would enable genome-targeted approaches to surgical planning, and targeted surveillance of patients with high-risk variants could be designed.

### Looking to the future – precision medicine and targeted therapy

Precision medicine utilising genomics and targeted therapy is touted as the future of clinical medicine, influencing screening, diagnosis, treatment, and follow-up management of patients.

Currently, the choice of imaging for PPGL may be influenced by the patient’s genome. Guidelines for radionuclide imaging of PPGL were updated jointly by the Society of Nuclear Medicine and Molecular Imaging (SNMMI) and the European Association of Nuclear Medicine (EANM) in 2019, which outline different nuclear imaging protocols for PPGL depending on tumour location and presence of germline variants (Taïeb *et al.* 2019). Their recommended first choice for imaging patients with *SDHx* mutations (regardless of tumour location) is [^68^Ga]SSA (gallium-68 labelled somatostatin analogue), with [^18^F]FDG (fluorine-18 labelled fluorodeoxyglucose) and [^18^F]FDOPA (fluorine-18 labelled fluorodihydroxyphenylalanine) as second-choice options, consistent with findings that SDH-deficient tumours have higher expression of somatostatin receptors compared to SDH-sufficient tumours (Elston *et al.* 2015).

In the future, therapy may be targeted towards specific germline variants, as discussed in detail in a recent review (Nölting *et al.* 2022). One area of research is in radiotherapeutics. Due to the high somatostatin receptor expression in *SDHx* tumours, researchers are investigating the use of peptide receptor radionuclide therapy (PRRT), such as [^117^Lu]DOTATATE (lutetium-177 labelled DOTA-Tyr3-octreotate) – a common treatment option for other neuroendocrine tumours – for progressive or metastatic PPGL (Lindenberg *et al.* 2019, Vyakaranam *et al.* 2019). For *SDHA* PPGL, chemotherapies that target the pseudohypoxia pathway are being explored, with various clinical trials investigating the use of tyrosine kinase inhibitors, such as sunitinib and lenvatinib, as antiangiogenic therapy for *SDHx* PPGL. Notably, a phase II clinical trial of sunitinib demonstrated the greatest benefit in patients with *SDHA, SDHB,* or *RET* germline PVs (O’Kane *et al.* 2019). Targeted therapies are also being investigated for use in combination with more classical chemotherapy regimens; for instance, one case report demonstrated the successful use of high-dose propranolol (3 mg/kg/day), hypothesised to have antiangiogenic properties through decreasing HIF-inducing transcription targets, in combination with temozolomide in treating *SDHA* metastatic paraganglioma (Díaz-Castellanos *et al.* 2017). Therapies targeting reactive oxygen species (ROS) and NAD+/PARP DNA repair have shown promise for *SDHB* tumours (Pang *et al.* 2018, Hadrava Vanova *et al.* 2021); and may become possible therapeutic options for *SDHA* tumours in the future.

Recently, certain microRNA (miRNA) and long noncoding RNA (lncRNA) signatures have been associated with metastasis-free survival in *SDHx* deficient PPGL (Ghosal *et al.* 2022); suggesting a future of targeted monitoring of disease post-treatment, guided by genomics.

## Clinical genetics

### Disease penetrance

One of the factors complicating current clinical management of patients found to carry *SDHA* PVs is the low penetrance of *SDHA*-related PPGL. Penetrance can be estimated either from family or cohort studies, or by inference from the prevalence of *SDHA* variants in large population databases, such as gnomAD (https://gnomad.broadinstitute.org) (Maniam *et al.* 2018). Using the former approach, penetrance of *SDHA* PVs was estimated at 10% (95% confidence interval, 0% to 21%) by age 70 (van der Tuin *et al.* 2018). By comparison, penetrance for *SDHB* and paternally inherited *SDHD* PV carriers was estimated to be as high as 44% and 90% by age 70, respectively (Fishbein & Nathanson 2012, Rijken *et al.* 2018). Using Bayesian approaches based on the prevalence of *SDHA* PVs in population genetic databases, penetrance for *SDHA* PVs was estimated at 1.0–4.9% (Maniam *et al.* 2018) or 1.7% (0.8–3.8%) (Benn *et al.* 2018). Notably, most *SDHA* PVs have been found to have been inherited from a clinically unaffected parent (van der Tuin *et al.* 2018).

It is thought the rarity and low penetrance of *SDHA*-related tumours are due to the low frequency of LOH at 5p15, where *SDHA* is located, compared to the more common losses at 1p36 and 11q23 loci where *SDHB* and *SDHD* are located (Burnichon *et al.* 2010). Traditionally, mutation rates throughout the genome were thought to be random, but recent research has shown epigenome-associated mutation is biased in a manner that may provide a protective effect to essential loci (Monroe *et al.* 2022). Although speculative, this process may in part explain the reduced penetrance of *SDHA* germline PVs, perhaps through protecting against loss of heterozygosity in *SDHA*. There have been various conjectures as to the coexistence of low penetrance yet high metastatic potential of *SDHA*. Possibly, loss of SDHA causes such a severe impact on SDH function as to be usually impermissible to cell survival, butt when *SDHA*-deficient cells do manage to survive, perhaps via additional somatic genetic or epigenetic alterations, the disease process is particularly aggressive (Jha *et al.* 2019).

### Variant pathogenicity

Establishing the pathogenicity of *SDHA* variants is essential for appropriate patient management and genetic counseling. When a germline *SDHA* variant is found in a patient presenting with PPGL, a careful assessment by an expert geneticist is required to determine whether the variant is pathogenic or not (Richards *et al.* 2015). Loss-of-function variants (i.e. variants predicted to lead to premature transcript termination, splicing variants, or large deletions) are afforded higher weighting in these calculations; as such, the assignment of pathogenicity for missense variants can be challenging. Additional evidence may be provided by functional studies (such as loss of staining of SDHA IHC, or finding elevated tumoral succinate (Richter *et al.* 2023). Segration of variants with disease across multiple family members is rarely found for a low penetrance disorder such as *SDHA*-related PPGL. *In silico* tools such as REVEL (rare exome variant ensemble learner), Polyphen-2 (polymorphism phenotyping version 2), or SIFT (sorting intolerant from tolerant) have not yet been validated for SDHA.

### Predictive *SDHA* variant testing and management of asymptomatic *SDHA* variant carriers

Identification of the genetic basis of PPGL allows for targeted testing of relatives who may carry the PV, with subsequent surveillance of carriers to enable early detection of any cancers that may arise. However, due to the low penetrance of *SDHA* variants and oftentimes unknown pathogenicity of variants, the case for testing and lifelong surveillance of mutation carriers—which can be a significant psychological burden—is weak compared with other genes with higher penetrance, such as *SDHB* and *SDHD.* In fact, some have argued against *SDHA* genetic testing of asymptomatic relatives (Maniam *et al.* 2018), and other groups have suggested a cautious approach when considering *SDHA* variant testing due to the low disease penetrance (Else & Fishbein 2018).

There are various factors to consider when approaching predictive *SDHA* testing and disease penetrance. Firstly, there is a higher-than-predicted background population prevalence for *SDHA* PVs, particularly the c.91C>T (p.Arg31*) variant. This has led to Bayesian analyses using population data which have estimated *SDHA* PV penetrance between 1.0–4.9% (Benn *et al.* 2018, Maniam *et al.* 2018). This low prevalence is also evidenced by family studies, such as the van der Tuin *et al.* (2018) study, which identified PPGL in only 1/56 non-index *SDHA* PV carriers with age-related penetrance for their non-index *SDHA* PV carriers estimated to be 0% at age 25, 2% at age 50 (95% confidence interval, 0–6%), and 10% at age 70 (95% confidence interval, 0–21%). The rarity of positive family history in index *SDHA* PPGL cases, as demonstrated in our analysis, is further suggestive of low disease penetrance. In view of this, recent UK guidelines suggest that predictive *SDHA* testing should only be offered to first-degree relatives of an affected proband, in the absence of a wider family history of *SDHA* disease, and only after thorough and transparent discussion with patients and their families regarding the low penetrance of disease and lack of clear evidence for the utility of surveillance were PVs to be identified (Hanson *et al.* 2023). Additionally, asymptomatic individuals without a personal or family history of *SDHA-*associated tumours who are incidentally found to carry an *SDHA* PV should not be offered tumour surveillance (nor their family), according to UK guidelines recommendations two and three (Hanson *et al.* 2023). In our clinical experience, a family history of metastatic disease commonly influences the family’s decision for predictive testing and subsequent surveillance, although this may be based on an emotive rather than scientific basis. As the understanding of *SDHA* in PPGL has improved, multiple guidelines have been published discussing predictive testing of relatives and their subsequent management, and these recommendations have been summarised in Table 3 (Papathomas *et al.* 2015, UK Cancer Genetics Group 2019, Wong *et al.* 2019, Amar *et al.* 2021, Hanson *et al.* 2023).

**Table 3:** Summary of recommendations from international guidelines on predictive germline *SDHA* variant testing of relatives of patients identified with *SDHA-*associated PPGL.

**Guideline**	**Recommendations**	
**UK Guidelines**(Hanson *et al.* 2023)	Predictive *SDHA* testing	Recommended in first-degree relatives**^a^**. Referral to clinical genetics is suggested.
	When to begin testing	From 10 years of age.
	Initial screening	As per UKCGG guidelines.
	Follow-up screening	As per UKCGG guidelines.
	Exit from screening	As per international consensus 2021.
**International Consensus**(Amar *et al.* 2021)	Predictive *SDHA* testing	Recommended (Grade A).
	When to begin testing	Between ages 10–15 (Grade A).
	Initial screening	Clinical: Blood pressure and symptoms and signs questionnaire. Biochemical: plasma or urinary metanephrines and normetanephrines (children) or plasma-free metanephrines and normetanephrines (adults). Imaging: MRI head and neck, thorax, abdomen, and pelvis (children) or MRI head and neck, abdomen and pelvis (Grade A), and PET-CT (Grade A) (adults).
	Follow-up screening	Yearly clinical examination, blood pressure measurement, and symptom questionnaire (Grade A). Biochemistry: at least every 2 years (child) or yearly (adult). Tests as per the initial screening (Grade A). Imaging: MRI every 2–3 years (Grade A).
	Exit from screening	From age 70, the imaging interval may be extended to every 5 years if asymptomatic and no previous tumour development (Grade B). Screening may be stopped from age 80 if asymptomatic (Grade A).
**UK Cancer Genetics Group (UKCGG)** (UK Cancer Genetics Group 2019)	Predictive *SDHA* testing	From age 10.
	When to begin testing	Biochemical screening from age 10. Radiological screening from age 15.
	Initial screening	Annual symptom review and clinical examination including blood pressure. Biochemistry: plasma metanephrines (or 24-hour urinary metanephrines) and 3-methoxytyramine. Radiological surveillance every 3–5 years: neck, thorax, abdomen, and pelvis, preferably with MRI.
	Follow-up screening	As per the initial screening.
	Exit from screening	Until 75 years as a minimum.
**Pediatric Guidelines**(Wong *et al.* 2019)	Predictive *SDHA* testing	May be offered from age 10.
	When to begin testing	Clinical exam and biochemical screening from 10 years. Radiological screening from 15 years.
	Initial screening	Annual biochemical testing (not discussed in detail). MRI head and neck every 2–3 years if asymptomatic and biochemistry normal.
	Follow-up screening	As per follow-up screening.
	Exit from screening	Not discussed.

**^a^**In the absence of a wider family history of *SDHA*-associated tumours.

Studies evaluating the efficacy of surveillance of *SDHA* variant carriers are limited. A study by Greenberg *et al.* (2020) diagnosed PPGL during follow-up in 1 out of 9 patients (11%) with known germline *SDHA* PVs. A case report in 2019 described a patient who was diagnosed with HNPGL following incidental identification of a germline *SDHA* PV on a gene panel for cardiomyopathy, arguing in support of initial screening of patients identified with *SDHA* PVs (White *et al.* 2019). Although the HNPGL was identified, the patient was managed conservatively due to underlying comorbidities and the asymptomatic nature of the disease. Larger longitudinal studies would be required to appropriately evaluate the efficacy of active surveillance compared to patient education to self-monitor for symptoms.

## Conclusions

Since identification in 2010, *SDHA* has become an increasingly recognised and important PPGL susceptibility gene, with *SDHA* variants accounting for up to 2.8% of patients with PPGL. Like other *SDHx* variants, *SDHA* PVs are thought to act through a pseudo-hypoxic drive, stimulating angiogenesis and cancer proliferation, with additional epigenetic effects influencing gene expression. Our review highlights that *SDHA*-associated PPGLs occur across a wide age range and include HNPGLs, abdominal PGLs, and phaeochromocytomas. While the true rate of metastasis and recurrence remains unclear, there appears to be an elevated metastatic risk with *SDHA* PVs, with bones and lymph nodes the most likely sites of metastatic spread. The penetrance of *SDHA* PVs is relatively low, and the decision to test and screen first-degree family members should ideally occur within expert family cancer centre settings.

Identification of the genetic basis of hereditary PPGL enables targeted care of patients and their families, foreshadowing an increasing personalisation of medical treatment in the future. Current management of *SDHA* carriers remains challenging due to the low penetrance yet elevated potential for aggressive disease. Recent international consensus guidelines will aid the management of patients and their families, with an interdisciplinary approach essential for the best outcomes.

## Supplementary Materials

Supplementary Methods

Supplementary Table 1-2

Supplementary Table 3. Likely benign SDHA variants excluded from analysis.

## Declaration of interest

The authors declare that there is no conflict of interest that could be perceived as prejudicing the impartiality of the research reported.

## Funding

This work did not receive any specific grant from any funding agency in the public, commercial, or not-for-profit sector.
