# Supplementary material for: SDHA-related phaeochromocytoma and paraganglioma: review and clinical management
Source: Endocr Relat Cancer. 2024 Sep 21;31(10):e240111. doi: 10.1530/ERC-24-0111 (PMC11466202; doi:10.1530/ERC-24-0111)
Supplement: Supplementary Methods [file supplementary_methods.pdf]

Methods - supplementary

Search strategy for compilation of *SDHA* PGL/PCC case series

A literature review was conducted to identify cases of previously reported *SDHA*-related PGL/PCC disease.

One researcher (AK) performed a search of the Medline® database to identify relevant reports published from inception to April 15, 2022. The search algorithm was based on combinations of various spellings and grammatical iterations of the terms "paraganglioma OR phaeochromocytoma" AND "*SDHA* OR succinate dehydrogenase subunit A". No limit was applied with respect to study type or language in the initial search. Reference lists of the final selection of studies were manually searched to identify additional relevant papers. A flow diagram for identification of studies has been produced (Supplementary Figure 1).

Studies were first screened by title for relevance, with subsequent abstract review. Articles where a full-text version or English-language version was not available were excluded. Only articles regarding human patients were included. Articles identified as potentially relevant were examined through review of the full text. Studies that fulfilled both eligibility criteria were included. Criteria (1), PGL/PCC tumours with a confirmed germline mutation in *SDHA*. Criteria (2), demographic information on the individual case including patient age and sex. All included studies were additionally searched for further characterization of cases, including location of the PGL/PCC, immunohistochemistry results, disease recurrence, metastatic disease, family history of *SDHA*-related PGL/PCC disease, and secretory nature of the tumour. In situations where identical patients were included in multiple studies, the case was recorded with the first publication.

Classification of *SDHA* variant status

SDHA variants were assessed for likelihood of pathogenicity using Varsome and the ClinVar database, and all variants were reviewed by our cancer genetics diagnostic laboratory team. Variants of 'likely benign' description (variant n=4) were excluded from analysis (see supplemental table 3 for details), while variants of unknown significance were separated from the primary analysis (Supplementary Figure 1).

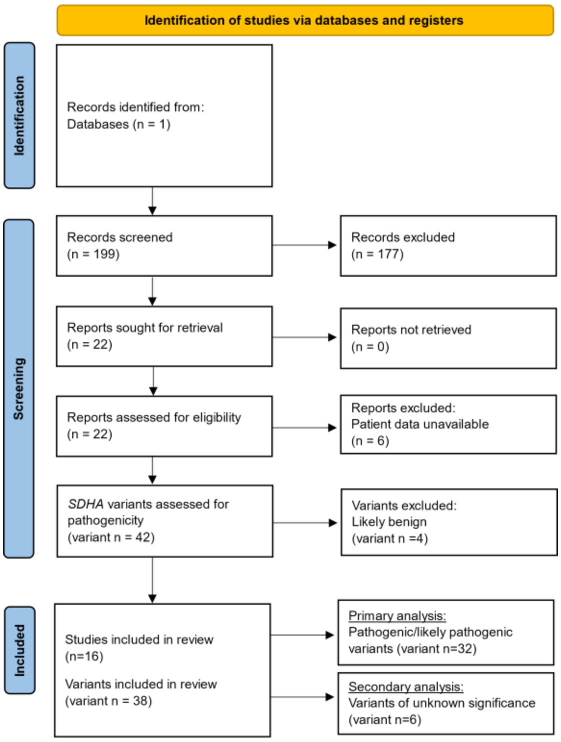

Supplementary Figure 1. Flow diagram of literature review for compilation of *SDHA* PGL/PCC case series.
