# Supplementary material for: SDHA-related phaeochromocytoma and paraganglioma: review and clinical management
Source: Endocr Relat Cancer. 2024 Sep 21;31(10):e240111. doi: 10.1530/ERC-24-0111 (PMC11466202; doi:10.1530/ERC-24-0111)
Supplement: Supplementary Table 1-2 [file supplementary_table_1-2.pdf]

Supplementary Table 1. Clinical information on individual patients diagnosed with PPGL in the literature.

Primary analysis (n=98)

Age	Gender	Location	Single/multiple	Germline variant	Protein change	SDHA IHC	SDHB IHC	Recurrence	Time to recurrence (years)	Metastases	Metastatic sites	Years to metastasis	Family History	Secreting	Reference	Patient number (from study)	Notes
48	F	PCC	Single	‡	‡	Negative	Negative			No			Nil	NA	Korpershoek 2011	132	See legend for explanation
63	F	pPGL - carotid	Single	c.1177G>A	p.Val393Met	Not tested	Not tested						Nil	Not reported	Bausch 2017	22	
58	M	PCC	Bilateral	c.1283>1298del	p.Gln428Profs*37	Not tested	Not tested						Nil	Not reported	Bausch 2017	23	
30	F	sPGL - pelvis	Single	c.1316G>A	p.Gly439Glu	Not tested	Not tested						Nil	Not reported	Bausch 2017	24	
53	F	sPGL - abdomen	Single	c.1534C>T	p.Arg512*	Not tested	Not tested	Yes	0.25	Yes	Bones, lymph nodes, lungs	Synchronous	Nil	NA, DOPA	Jha 2019	2	Metastatic paper
29	F	sPGL - abdomen	Single	c.5'UTR_3'UTR del		Not tested	Not tested	Yes	1.00	Yes	Bones, lymph nodes, lungs	1.67	Nil	NA	Jha 2019	8	Metastatic paper
20	F	pPGL - jugular	Single	c.1334C>T	p.Ser445Leu	Not tested	Not tested						Nil	Not reported	Bausch 2017	25	
47	F	PGL - thorax + pPGL - carotid	Multiple	c.1338delA	p.His447Metfs*23	Negative	Not tested			No			Nil	NA (PGL), DOPA (carotid body)	Tufton 2017	3	
48	F	HNPGL	Single	c.1338delA	p.His447Metfs*23	Not tested	Not tested			No			Nil	Non-secretory	Casey 2017 (May)		Frameshift
48	M	pPGL - carotid	Bilateral	c.1340A>G	p.His447Arg	Not tested	Not tested						Nil	Not reported	Bausch 2017	26	
49	M	pPGL - jugular	Single	c.1361C>A	p.Ala454Glu	Not tested	Not tested						Nil	Not reported	Bausch 2017	27	
18	F	PCC	Single	c.91C>T	p.Arg31*	Not tested	Not tested	Yes	36	Yes	Bones, lymph nodes	36	Nil	NA	Tufton 2017	9	Recurrence: PCC (same side) 36 years later with metastatic disase. Mets: sacrum, T10, para-aortic LN, paracaval LN
43	M	pPGL - carotid	Single	c.1432_1432+1del	Splice donor	Not tested	Not tested						Nil	Non-secretory	van der tuin 2018	8	
44	M	sPGL - abdomen	Single	c.1432_1432+1del	Splice donor	Not tested	Not tested						Nil	Not reported	Bausch 2017	29	
50	F	PCC	Single	c.91C>T	p.Arg31*	Not tested	Not tested			Yes	Not reported	10	Nil	NA	van der tuin 2018	24	
46	F	sPGL - abdomen	Single	c.91C>T	p.Arg31*	Not tested	Not tested	Yes	7.00	Yes	Bones, lymph nodes, lungs, liver	6.5	Nil	NA, DOPA	Jha 2019	9	Metastatic paper

11	F	pPGL - vagal	Single	c.91C>T	p.Arg31*	Not tested	Not tested	Yes	3	Yes	Bones, lymph nodes, lungs		1	Nil	Non-secretory	Jha 2019	1	Metastatic paper
27	M	sPGL - abdomen	Single	c.1753C>T	p.Arg585Trp	Negative	Negative			No				Nil	NA	Korpershoek 2011	291	Had this variant
34	F	PGL - unspecified	Single	c.1753C>T	p.Arg585Trp	Not tested	Not tested			No				Nil	Non-secretory	Casey 2017 (May)		
34	F	PGL - thorax	Single	c.1753C>T	p.Arg585Trp	Negative	Not tested			No				Nil	Non-secretory	Tufton 2017	7	The cases were all "index" cases, but there were 5 asymptomatic carriers identified through cascade screening of them
19	F	PCC	Single	c.1753C>T	p.Arg585Trp	Negative	Negative			No				Not reported	Not reported	Papathomas 2015	170	
26	F	sPGL - abdomen	Single	c.1754G>A	p.Arg585Gln	Negative	Negative			No				Not reported	NA	Currás-Freixes 2015	140	Identified through Lee 2020 review
32	F	sPGL - abdomen	Single	c.1765C>T	p.Arg589Trp	Negative	Negative			No				Nil	NA	Burnichon et al. (2010)	1	
31	F	sPGL - abdomen	Single	c.1765C>T	p.Arg589Trp	Negative	Negative			No				Not reported	Not reported	Papathomas 2015	103	
50	F	pPGL - jugular	Single	c.1766G>A	p.Arg589Gln	Not tested	Not tested							Nil	Not reported	Bausch 2017	30	
24	M	PCC	Single	c.1766G>A	p.Arg589Gln	Negative	Negative			No				Not reported	Not reported	Papathomas 2015	14	
60	M	pPGL - vagal	Single	c.1795-3C>G	Intronic variant	Not tested	Not tested							Nil	DOPA	van der tuin 2018	20	
66	M	PGL - thorax	Single	c.1A>C	p.Met1?	Not tested	Not tested			No				Nil	Not reported	Bausch 2017	1	
30	M	PCC	Single	c.1A>T	p.Met1?	Not tested	Not tested							Nil	Not reported	Bausch 2017	2	
20	F	sPGL - abdomen	Single	c.223C>T	p.Arg75*	Negative	Negative			No				Nil	Non-secretory	Welander 2013		
20	F	sPGL - abdomen	Single	c.223C>T	p.Arg75*	Not tested	Not tested							Nil	Not reported	Bausch 2017	10	
47	M	PCC	Single	c.223C>T	p.Arg75*	Not tested	Not tested							Nil	Not reported	Bausch 2017	11	
47	F	pPGL - jugular	Single	c.296A>G	p.His99Arg	Not tested	Not tested							Nil	Not reported	Bausch 2017	12	
27	F	pPGL - carotid	Single	c.2T>G	p.Met1?	Not tested	Not tested							Nil	Not reported	Bausch 2017	3	
36	F	pPGL - thyroid	Single	c.394T>C	p.Trp132Arg	Not tested	Not tested			No				Nil	Non-secretory	von Dobschuetz 2015	3	
34	M	PCC	Single	c.3G>C	p.Met1?	Not tested	Not tested							Nil	Not reported	Bausch 2017	4	
43	F	PGL - thorax	Single	c.923C>T	p.Thr308Met	Not tested	Not tested			Yes	Not reported	Synchronous		Nil	Yes	Casey 2017 (May)		

15	F	sPGL - abdomen	Single	c.457-1G>A		Not tested	Not tested			No			Not reported	Non-secretory	Currás-Freixes 2015	155	
26	F	pPGL - jugular	Single	c.457-1G>A		Not tested	Not tested						Nil	Not reported	Bausch 2017	13	
Not reported	F	sPGL - abdomen	Single	c.563G>A	p.Arg188Gln	Not tested	Not tested	Yes	2.00	No			Not reported	Not reported	Greenberg 2020	42	
17	M	sPGL - abdomen	Single	c.566G>A	p.Cys189Tyr	Not tested	Not tested						Nil	Not reported	Bausch 2017	14	
53	M	PGL - thorax	Single	c.1334C>T	p.Ser445Leu	Not tested	Not tested	Yes	3.17	Yes	Bones	4	Nil	NA	Jha 2019	5	Metastatic paper
49	M	pPGL - vagal + pPGL - carotid	Multiple	c.667delG	p.Asp223fs	Not tested	Not tested						Nil	Non-secretory	van der tuin 2018	21	
53	M	pPGL - jugular	Single	c.778G>A	p.Gly260Arg	Positive	Not tested						Nil	Not reported	Bausch 2017	17	SDHA tested in Van der tuin 2018
33	M	sPGL - pelvis	Single	c.820G>A	p.Gly274Ser	Not tested	Not tested						Nil	Not reported	Bausch 2017	18	
53	M	PGL - thorax	Single	c.1334C>T	p.Ser445Leu	Not tested	Not tested			Yes	Bones	4.5	Nil	NA, A	Díaz-Castellanos 2017	1	
38	M	pPGL - jugular tympanic	Single	c.91C>T	p.Arg31*	Not tested	Not tested						Nil	DOPA	van der tuin 2018	11	
28	M	PCC	Single	c.1361C>A	p.Ala454Glu	Not tested	Not tested			Yes†	Not reported	Not reported	Nil	Not reported	Bausch 2017	28	4/34 of this paper's cases were classified as malignant, however information is only provided for this one patient. To reflect this, 3 extra cases of metastatic disease were included in calculations for metastatic cases.
23	M	sPGL - testis	Single	c.1534C>T	p.Arg512*	Not tested	Not tested			Yes	Not reported	3	Yes - *SDHA* HNPGL	NA, DOPA	van der tuin 2018	27	
23	M	sPGL - abdomen	Single	c.1534C>T	p.Arg512*	Heterogenous	Negative			Yes	Para-aortic LN	Not reported	Not reported	Not reported	Papathomas 2015	182	SDHA staining was defined as heterogenous: SDHA staining was negative in the centre of the tumour but positive on the periphery. For the purposes of this study, it has been defined as negative staining due to the centre of the tumour being negative.
30	M	pPGL - carotid	Single	c.91C>T	p.Arg31*	Not tested	Not tested						Nil	NA	van der tuin 2018	6	
61	F	pPGL - carotid	Single	c.91C>T	p.Arg31*	Not tested	Not tested						Nil	NA	van der tuin 2018	13	

18	M	pPGL - jugular	Single	c.91C>T	p.Arg31*	Not tested	Not tested						Nil	NA	van der tuin 2018	18	
22	M	sPGL - abdomen	Single	c.91C>T	p.Arg31*	Not tested	Not tested						Nil	NA	van der tuin 2018	30	
71	M	sPGL - abdomen	Single	c.457-1G>A		Negative	Negative	Not reported		Yes	Bone	Synchronous	Not reported	NA	Currás-Freixes 2015	195	Only bone listed in table as metastatic site
57	M	sPGL - abdomen + HNPGL (on follow-up)	Multiple	c.91C>T	p.Arg31*	Positive	Negative	Yes	0.58	Yes	Lymph node	0.58	Nil	A, DOPA	Jha 2019/Wurth 2021	4	Metastatic paper
60	M	sPGL - abdomen	Single	c.91C>T	p.Arg31*	Not tested	Not tested						Nil - RCC (non-SDHA)	NA	van der tuin 2018	26	
20	M	sPGL - abdomen	Single	c.91C>T	p.Arg31*	Not tested	Not tested	Yes	2.58	Yes	Bones and lymph nodes	Synchronous	Nil	NA	Jha 2019	6	Metastatic paper
50	M	sPGL - abdomen	Single	c.91C>T	p.Arg31*	Not tested	Not tested						Nil	NA, DOPA	van der tuin 2018	28	
14	M	sPGL - abdomen	Single	c.91C>T	p.Arg31*	Not tested	Not tested	Yes	0.17	Yes	Bones, lymph nodes	Synchronous	Nil	NA	Jha 2019	3	Metastatic paper
56	M	PCC	Single	c.91C>T	p.Arg31*	Not tested	Not tested	Unknown		Yes	Bones, lymph nodes, lungs, liver	10	Nil	NA	Jha 2019	7	Metastatic paper
56	M	HNPGL	Single	c.91C>T	p.Arg31*	Not tested	Not tested			No			Nil	Non-secretory	Casey 2017 (May)		
68	M	sPGL - abdomen	SIngle	c.91C>T	p.Arg31*	Not tested	Not tested			No			Nil	Non-secretory	Tufton 2017	11	Metastatic potential (vascular invasion)
44	M	sPGL - abdomen	Single	c.91C>T	p.Arg31*	Not tested	Not tested	Yes	0.67	Yes	Bones, lymph nodes, lungs	Synchronous	Nil	NA, A, DOPA	Jha 2019	10	Metastatic paper
43	F	pPGL - carotid	Single	c.91C>T	p.Arg31*	Not tested	Not tested						Nil	Non-secretory	van der tuin 2018	1	
38	M	pPGL - vagal	Single	c.91C>T	p.Arg31*	Not tested	Not tested						Nil	Non-secretory	van der tuin 2018	2	
81	F	pPGL - vagal	Single	c.91C>T	p.Arg31*	Not tested	Not tested						Nil	Non-secretory	van der tuin 2018	3	
35	F	pPGL - carotid	Multiple (unilateral)	c.91C>T	p.Arg31*	Not tested	Not tested						Nil	Non-secretory	van der tuin 2018	4	
48	F	pPGL - carotid + pPGL - jugular	Multiple	c.91C>T	p.Arg31*	Not tested	Not tested						Nil	Non-secretory	van der tuin 2018	5	
56	M	pPGL - carotid	Single	c.91C>T	p.Arg31*	Not tested	Not tested						Nil	Non-secretory	van der tuin 2018	7	
26	F	pPGL - carotid	Multiple	c.91C>T	p.Arg31*	Not tested	Not tested	Yes					Nil	Non-secretory	van der tuin 2018	9	Multiple with recurrence
58	F	pPGL -	Single	c.91C>T	p.Arg31*	Not tested	Not						Nil	Non-	van der	14	

		jugular tympanic					tested					secretory	tuin 2018		
53	F	pPGL - vagal	Single	c.91C>T	p.Arg31*	Not tested	Not tested				Nil	Non-secretory	van der tuin 2018	15	
42	F	pPGL - vagal	Single	c.91C>T	p.Arg31*	Not tested	Not tested				Nil	Non-secretory	van der tuin 2018	16	
48	M	pPGL - carotid	Single	c.91C>T	p.Arg31*	Not tested	Not tested				Nil	Non-secretory	van der tuin 2018	19	
70	M	PCC	Single	c.91C>T	p.Arg31*	Not tested	Not tested				Nil	Non-secretory	van der tuin 2018	25	
72	F	pPGL - carotid	Single	c.91C>T	p.Arg31*	Not tested	Not tested	No			Nil	Non-secretory	White 2019	1	Case identified from screening in an asymptomatic SDHA germline mutation carrier (identified on cardiomyopathy panel). Diagnosis was made on location, clinical history and MRI scan - no histololgy was obtained as the tumour was not resected.
53	F	pPGL - vagal	Single	c.91C>T	p.Arg31*	Not tested	Not tested				Possible - RCC (SDHA unknown)	Non-secretory	van der tuin 2018	17	
23	M	pPGL - carotid	Single	c.91C>T	p.Arg31*	Not tested	Not tested				Yes - *SDHA* GIST	Non-secretory	van der tuin 2018	10	
55	F	PGL - thorax	Single	c.91C>T	p.Arg31* ‡	Negative	Negative	No			Nil	Not reported	Korpershoek 2011	161	Had this variant
33	M	sPGL - abdomen	Single	c.91C>T	p.Arg31*	Not tested	Not tested	No			Nil	Not reported	Casey 2017 (May)		
36	F	pPGL - thyroid	Single	c.91C>T	p.Arg31*	Not tested	Not tested	No			Nil	Not reported	Tufton 2017	1	
15	M	PCC	Single	c.91C>T	p.Arg31*	Not tested	Not tested				Nil	Not reported	Bausch 2017	5	
20	F	pPGL - carotid	Single	c.91C>T	p.Arg31*	Not tested	Not tested				Nil	Not reported	Bausch 2017	7	
37	F	pPGL - carotid	Single	c.91C>T	p.Arg31*	Not tested	Not tested				Nil	Not reported	Bausch 2017	8	
34	F	pPGL - jugular	Single	c.91C>T	p.Arg31*	Not tested	Not tested				Nil	Not reported	Bausch 2017	9	
36	M	sPGL - abdomen	Single	c.91C>T	p.Arg31*	Not tested	Not tested				Yes	Not reported	Bausch 2017	6	
36	M	sPGL - abdomen	Single	c.91C>T	p.Arg31*	Not tested	Not tested	Yes	Not reported	Synchronous	Nil	NA, DOPA	van der tuin 2018	29	
45	M	sPGL - abdomen	Single	c.91C>T	p.Arg31*	Not tested	Not tested	No			Nil	Yes	Casey 2017 (May)		
zz	F	PCC	Single	c.91C>T	p.Arg31*	Not tested	Not tested	No			Nil	Yes	Casey 2017 (May)		

23	M	sPGL - abdomen	Single	c.91C>T	p.Arg31*	Not tested	Negative	No		Yes	Bone	23	Nil	With metastatic presentation - NA	Casey 2017 (Aug)	1	Single case. Met to rib 8
33	F	pPGL - vagal	Single	c.91C>T ‡	p.Arg31* ‡	Negative	Negative			No			Nil	Not reported	Korpershoek 2011	162	
45	M	pPGL - carotid	Single	c.91C>T ‡	p.Arg31* ‡	Negative	Negative			No			Nil	Not reported	Korpershoek 2011	187	
41	M	sPGL - bladder	Single	c.91C>T ‡	p.Arg31* ‡	Negative	Negative			Yes	Local LN	Not reported	Nil	NA, A	Korpershoek 2011	146	
52	M	PCC + HNPGL	Multiple	c.923C>T	p.Thr308Met	Not tested	Not tested			No			Nil	Yes	Casey 2017 (May)		
46	M	sPGL - abdomen	Single	c.923C>T	p.Thr308Met	Not tested	Not tested	Yes	1	Yes	Bone, adrenals	16	Nil	NA	Tufton 2017	8	Multiple recurrence. Mets: L4 and adrenals
46	F	pPGL - jugular	Single	c.940G>A	p.Glu314Lys	Not tested	Not tested						Nil	Not reported	Bausch 2017	20	
40	F	pPGL - carotid	Single	c.985C>T	p.Arg329*	Not tested	Not tested						Yes - *SDHA* RCC	Non-secretory	van der tuin 2018	12	

Legend

‡ The p.Arg31* variant was identified in four of these patients. One patient had inadequate DNA quality for assessment of variants, however, it is unclear which patient this was.

† Bausch et al., 2017 note malignancy in four patients, however, malignancy was only defined for patient 28. 4/34 of their patients were considered to have malignancy for statistical analysis.

Secondary Analysis – variants of uncertain significance (n=9)

Age	Gender	Location	Single/multiple	Germline variant	Protein change	SDHA IHC	SDHB IHC	Recurrence	Time to recurrence (years)	Metastases	Metastatic sites	Years to metastasis	Family history	Secreting	Reference	Patient number (from study)	Notes
43	M	pPGL - carotid	Single	c.622T>C	p.Ser208Pro	Not tested	Not tested						Nil	Not reported	Bausch 2017	15	
64	M	PCC	Single	c.629G>A	p.Arg210Gln	Not tested	Not tested						Nil	Not reported	Bausch 2017	16	
24	M	PCC	Single	c.1115C>G	p.Pro372Arg	Not tested	Not tested						Nil	Not reported	Bausch 2017	21	
62	M	PCC + sPGL -	Multiple	c.1273G>A	p.Val425Met	Not tested	Not tested			No			Nil	Yes	Casey 2017 (May)		

		abdomen													
65	F	pPGL - carotid	Single	c.1799G>A	p.Arg600Gln	Not tested	Not tested				Nil	Not reported	Bausch 2017	31	
49	M	sPGL - abdomen	Multiple	c.1799G>A	p.Arg600Gln	Not tested	Not tested				Nil	Not reported	Bausch 2017	32	
42	F	pPGL - vagal	Single	c.1799G>A	p.Arg600Gln	Not tested	Not tested				Nil	Not reported	Bausch 2017	33	
37	F	pPGL - thyroid	Single	c.1799G>A	p.Arg600Gln	Not tested	Not tested	Yes	12	No	Nil	Non-secretory	von Dobschuetz 2015	4	Tumour relapse/recurrence 12 years after thyroidectomy
46	F	pPGL - carotid	Single	c.1865G>A	p.Trp622*	Negative	Negative			No	Nil	Non-secretory	Dwight 2013	1	
