# Supplementary material for: SDHA-related phaeochromocytoma and paraganglioma: review and clinical management
Source: Endocr Relat Cancer. 2024 Sep 21;31(10):e240111. doi: 10.1530/ERC-24-0111 (PMC11466202; doi:10.1530/ERC-24-0111)
Supplement: Supplementary Table 3. Likely benign SDHA variants excluded from analysis. [file supplementary_table_3.pdf]

Supplementary Table 1. Clinical information on individual patients diagnosed with PPGL in the literature. Please see separate file (large).

Supplementary Table 2. Clinical features of 9 patients previously reported in the literature with phaeochromocytoma (PCC) and paraganglioma (PGL) associated with *SDHA* variants of uncertain significance (VUS)

* n/N: n represents the number of patients with the characteristic, while N represents the number of patients for which the information was available.

† Included one case which demonstrated heterogeneous SDHA staining, with no SDHA staining in the tumour core

**Feature**		***SDHA*-related PCC/PGL**
**Mean age at diagnosis,** y (SD)		48.0
**Gender,** n/N (%)*	Male	5/9 (56%)
	Female	4/9 (44%)
**Single PGL at presentation,** (%)		78.0
**Location,** n (% of 10 PPGL‡)	PCC	3
	HNPGL - carotid	3
	HNPGL - vagal	1
	HNPGL - thyroid	1
	TAPGL - abdomen	2
**Immunohistochemistry,** n/N (%)*	SDHA negative †	1/1 (100%)
	SDHB negative	1/1 (100%)
**Recurrent disease**	Recurrence at surgical site, n	1
	Median time to recurrence, y	12
**Metastatic disease,** n		0
**Biochemical status,** n	Biochemically active	1
	Biochemically silent	2
	Unknown profile	6
**Family history of PGL/PCC or *SDHA*-related tumours**, n		0

‡ Adjusted for patients with multiple tumour types.

Supplementary Table 3. Likely benign *SDHA* variants excluded from analysis.

SDHA variant	Varsome analysis	ClinVar status
c.133G>A	LB[α] (2P-6B)(BP4strong, BP6mod, PM2sup, PP2sup)	Uncertain significance (7); benign (2); likely benign (6)†
c.136A>G	LB (2P-4B)(BP4strong, PM2sup, PP2sup)	Uncertain significance (3); likely benign (5)
c.830C>T	LB (2P-3B)(BP6mod, BP4sup, PM2sup, PP2sup)	Uncertain significance (8); Benign (1); Likely benign (2)
c.1979C>G	LB (2P-4B)(BP4mod, BP6mod, PM2sup, PP2sup)	Uncertain significance (8); Benign (2); Likely benign (3)

α Varsome classification: Likely Benign (LB)

† Where ClinVar interpretation was conflicting, the various predicted effects with corresponding number of reports (X) have been shown.
